# Blood Transfusion, All-Cause Mortality and Hospitalization Period in COVID-19 Patients: Machine Learning Analysis of National Health Insurance Claims Data

**DOI:** 10.3390/diagnostics12122970

**Published:** 2022-11-28

**Authors:** Byung-Hyun Lee, Kwang-Sig Lee, Hae-In Kim, Jae-Seung Jung, Hyeon-Ju Shin, Jong-Hoon Park, Soon-Cheol Hong, Ki Hoon Ahn

**Affiliations:** 1 Department of Internal Medicine, Korea University Anam Hospital, Seoul 02841, Republic of Korea; 2 Korea University Anam Hospital Bloodless Medicine Center, Seoul 02841, Republic of Korea; 3 AI Center, Korea University Anam Hospital, Seoul 02841, Republic of Korea; 4 School of Industrial Management Engineering, Korea University, Seoul 02841, Republic of Korea; 5 Department of Obstetrics and Gynecology, Korea University Anam Hospital, Seoul 02841, Republic of Korea; 6 Department of Thoracic and Cardiovascular Surgery, Korea University Anam Hospital, Seoul 02841, Republic of Korea; 7 Department of Anesthesiology and Pain Medicine, Korea University Anam Hospital, Seoul 02841, Republic of Korea; 8 Department of Orthopedic Surgery, Korea University Anam Hospital, Seoul 02841, Republic of Korea

**Keywords:** blood transfusion, mortality, hospitalization, COVID-19, machine learning

## Abstract

This study presents the most comprehensive machine-learning analysis for the predictors of blood transfusion, all-cause mortality, and hospitalization period in COVID-19 patients. Data came from Korea National Health Insurance claims data with 7943 COVID-19 patients diagnosed during November 2019–May 2020. The dependent variables were all-cause mortality and the hospitalization period, and their 28 independent variables were considered. Random forest variable importance (GINI) was introduced for identifying the main factors of the dependent variables and evaluating their associations with these predictors, including blood transfusion. Based on the results of this study, blood transfusion had a positive association with all-cause mortality. The proportions of red blood cell, platelet, fresh frozen plasma, and cryoprecipitate transfusions were significantly higher in those with death than in those without death (*p*-values < 0.01). Likewise, the top ten factors of all-cause mortality based on random forest variable importance were the Charlson Comorbidity Index (53.54), age (45.68), socioeconomic status (45.65), red blood cell transfusion (27.08), dementia (19.27), antiplatelet (16.81), gender (14.60), diabetes mellitus (13.00), liver disease (11.19) and platelet transfusion (10.11). The top ten predictors of the hospitalization period were the Charlson Comorbidity Index, socioeconomic status, dementia, age, gender, hemiplegia, antiplatelet, diabetes mellitus, liver disease, and cardiovascular disease. In conclusion, comorbidity, red blood cell transfusion, and platelet transfusion were the major factors of all-cause mortality based on machine learning analysis. The effective management of these predictors is needed in COVID-19 patients.

## 1. Introduction

Since its outbreak in December 2019, coronavirus disease 2019 (COVID-19) caused by the severe acute respiratory syndrome coronavirus-2 spread rapidly, leading to a public health crisis [1]. Moreover, the COVID-19 outbreak dramatically increased global mortality [2,3]. Various clinical factors, including gender (male), age, and comorbidities (obesity, chronic kidney disease, cancer, diabetes mellitus, lung disease, etc.), are related to mortality and prognosis in patients with COVID-19 [4,5]. In addition, anemia is prevalent and associated with long hospital stays and poor clinical conditions in patients with COVID-19 [6]. Several studies evaluating the association between anemia and clinical outcomes of COVID-19 have shown that patients with anemia have higher mortality and worse prognosis [7,8,9]. However, other studies have not shown a negative association between anemia and the prognosis of patients with COVID-19 [10,11]. Thus, the effects of anemia on COVID-19 are debatable.

The COVID-19 pandemic had a significant impact on blood transfusions [12]. Concurrently, the pandemic had a substantial impact on supply of blood owing to a reduction in blood donation [12,13]. Studies have shown that many patients with COVID-19 do not require blood transfusion [12,14]. In a previous study, only one third of the patients required transfusion due to anemia (nonbleeding), and very few patients required platelet (PLT) or fresh frozen plasma (FFP) transfusion [12]. However, the literature investigating the prevalence and clinical effects of blood transfusion in patients with COVID-19 is limited. A previous study has reported that red blood cell (RBC) transfusion predicts overall mortality, and the mortality rate is directly proportional to the number of RBC units transfused [15]. Thus, RBC transfusion may be a prognostic predictor for adverse outcomes in patients with COVID-19; however, the clinical implications of blood transfusion in patients with COVID-19 remain unclear.

This study presents the most comprehensive machine learning analysis for the predictors of blood transfusion, all-cause mortality, and hospitalization period in COVID-19 patients, using a population-based cohort of 7943 participants from the Korean National Health Insurance Database (NHID) and the richest collection of 28 predictors, such as demographic/socioeconomic determinants, comorbid conditions, and disease information. All medical institutions mandatorily enter into a contract with the national government, and all prescriptions, orders, and diagnostic codes are computerized and recorded in the NHID. We analyzed the associations of blood transfusion in patients with COVID-19 with comorbidities. Further, we investigated the role of potential risk factors for all-cause mortality and hospitalization period in transfused and non-transfused patients with COVID-19. This study showed the overall implications of blood transfusion in patients with COVID-19.

## 2. Materials and Methods

### 2.1. Participants and Variables

This study used population data. Data of 7943 patients with COVID-19 diagnosed between 1 November 2019 and 31 May 2020 were obtained from the NHID. The NHID covers 52 million residents (nearly all residents) in Korea. All patients with any medical visit encoded as COVID-19 during the study period were included. The data size (7943) exceeds the minimum size to have desired properties with the 95% confidence interval and the 5% margin of error (156). The dependent variables were all-cause mortality and hospitalization period (days). This study considered 28 independent variables from the existing literature [1,2,3,4,5,6,7,8,9,10,11,12,13,14,15]: gender (male), socioeconomic status (insurance fee), Charlson Comorbidity Index (CCI) score and its components, transfusion history (RBC, PLT, FFP, and cryoprecipitate), medication history (anti-platelet agents, iron, and tranexamic acid), and other information (anemia and thrombocytopenia). The CCI has been developed as a weighted index to predict the risk of death within 1 year of hospitalization in patients with specific comorbid conditions [16]. Each condition is assigned a weight from 1 to 6, based on the estimated 1-year mortality hazard ratio, and these weights are summed to produce the comorbidity score [16]. In the present study, comorbidities were defined using the Anatomical Therapeutic Chemical (ATC)/International Classification of Diseases, Tenth Revision (ICD-10) codes. The CCI score ranged between 0 and 37, and was calculated as follows: age (0 to 4 points), myocardial infarction (I20–I25, 1 point), congestive heart failure (M30–M36, 1 point), peripheral vascular disease (K25–K28, 1 point), cardiovascular disease (I60–I69, 1 point), dementia (F00–F09, 1 point), chronic obstructive pulmonary disease (J41–J44, 1 point), connective tissue disease (M30–M36, 1 point), peptic ulcer disease (K25–K28, 1 point), liver disease (K70–K77, 1 or 3 points), diabetes mellitus (E10–E14, 1 or 2 points), hemiplegia (G80–G83, 2 points), chronic kidney disease (N18, 2 points), solid tumor (C00–C80, 2 or 6 points), leukemia (C91–C95, 2 points), lymphoma (C81–C90, 2 points), and AIDS (B20–B24, 6 points). The ATC/ICD-10 codes of transfusion history, medication history, and other information were RBC (X2021, X2022, X2031, X2032, X2091, X2092, X2111, X2112, X2512), PLT (X2081, X2082, X2121, X2122, X2501, X2511, X2513), FFP (X2041, X2042, X2051, X2052), and cryoprecipitate (X2061, X2062); antiplatelet agents (B01A), iron (B03A), and tranexamic acid (B02AA02); and anemia (D50–D53, D55–D59, D60–D64) and thrombocytopenia (D65, D69.3, D69.5, D69.6), respectively.

### 2.2. Analysis

The *t*-test was used to evaluate the associations of blood transfusion with CCI and all-cause mortality in patients with COVID-19. Next, random forest analysis for variable importance was performed to investigate the main factors of all-cause mortality and hospitalization period in patients with COVID-19 and to test their associations with transfusion history and other variables. A random forest in this study created 500 training sets, trained 500 decision trees, and made predictions with a majority vote. Random forest analysis for variable importance (the node impurity (GINI) decrease from the creation of a branch on a certain predictor) measures the importance of an independent variable for predicting the dependent variable. Indeed, logistic regression and random forest were employed to analyze the effects of blood transfusion on all-cause mortality and the hospitalization period for various subgroups (i.e., RBC, PLT, FFP, and cryoprecipitate transfusions). The results of these subgroup analyses are reported in the supplementary tables. The data were split into training and validation sets at a 70:30 ratio. The criteria for the validation of the trained models were (1) precision for the prediction of all-cause mortality, that is, the ratio of correct predictions among all cases, and (2) the mean absolute percentage error for the prediction of the hospitalization period (here, the error was the difference between the actual and predicted values of the hospitalization period) [17]. The statistical analyses were performed using R-Studio 1.3.959 (R-Studio Inc., Boston, MA, USA).

### 2.3. Ethics Statement

This retrospective study complied with the tenets of the Helsinki Declaration and was approved by the Institutional Review Board (IRB) of Korea University Anam Hospital on 10 August 2020 (2020AN0367). Informed consent was waived by the IRB, given that data were deidentified.

## 3. Results

**Figure 1:**
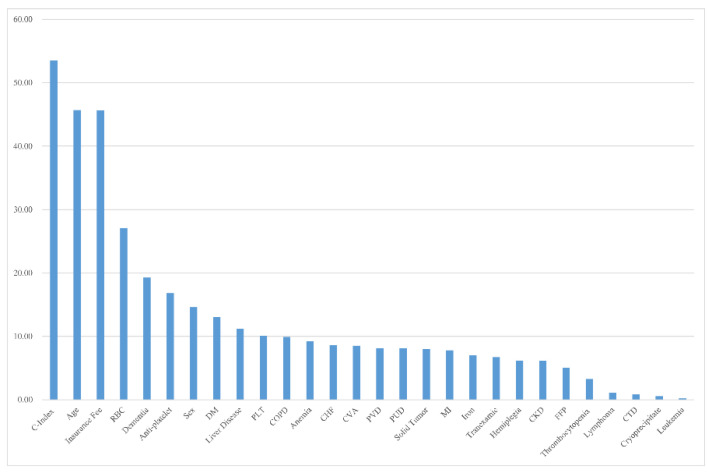
Random forest variable importance for death. Abbreviations: CHF, congestive heart failure; C-index, Charlson Comorbidity Index; CKD, chronic kidney disease; COPD, chronic obstructive pulmonary disease; CTD, connective tissue disease; CVA, cardiovascular accident; DM, diabetes mellitus; FFP, fresh frozen plasma; MI, myocardial infarction; PLT, platelet; PUD, peptic ulcer disease; PVD, peripheral vascular disease; RBC, red blood cell.

**Figure 2:**
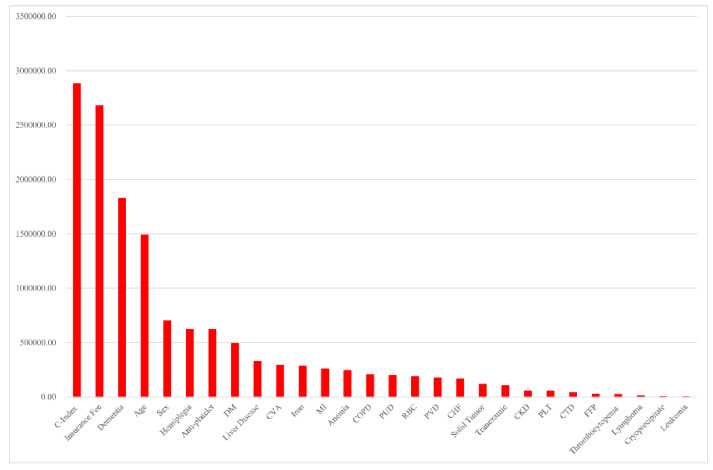
Random forest variable importance for hospitalization period. Abbreviations: CHF, congestive heart failure; C-index, Charlson Comorbidity Index; CKD, chronic kidney disease; COPD, chronic obstructive pulmonary disease; CTD, connective tissue disease; CVA, cardiovascular accident; DM, diabetes mellitus; FFP, fresh frozen plasma; MI, myocardial infarction; PLT, platelet; PUD, peptic ulcer disease; PVD, peripheral vascular disease; RBC, red blood cell.

Descriptive statistics for the 7943 patients with COVID-19 are summarized in Table 1 and Table 2. The median hospitalization period was 33 days and the median CCI, age, and socioeconomic status scores were 1, 4, and 10, respectively. Age was given with a range of 0–8 in the original data. Likewise, socioeconomic status ranged from 0 (highest health insurance payment) to 20 (lowest health insurance payment) in the original data. The proportion of those who died was 3.08% (n = 245). The proportions of patients with RBC, PLT, FFP, and cryoprecipitate transfusions were 2.29% (182), 0.65% (52), 0.43% (34), and 0.05% (4), respectively. The proportions of patients with gender (male), cardiovascular disease, diabetes mellitus, dementia, hemiplegia, liver disease, and antiplatelet medications were 60.00% (4766), 5.09% (404), 15.35% (1219), 6.48% (515), 1.13% (90), 17.55% (1394), and 12.07% (959), respectively. Blood transfusion was positively associated with the CCI score. The proportions of patients with RBC, PLT, and FFP transfusions were significantly higher in Group 1 (high CCI score) than in Group 2 (low CCI score) (Table 3). Blood transfusion was also positively associated with all-cause mortality. The proportions of patients with RBC, PLT, FFP, and cryoprecipitate transfusions were significantly higher in patients who died than in survivors (Table 4). Indeed, the proportion of patients who received RBC transfusion was significantly higher among those who died than among survivors (Table S1-1 (Group 1) and Table S1-2 (Group 2), supplementary tables). *p*-values for the test of their equality were <0.05, as reported in the notes of Tables S1-1 and S1-2. Likewise, blood transfusion was the major determinant of all-cause mortality and hospitalization period in patients with COVID-19 based on random forest variable importance. The top ten factors of all-cause mortality were CCI score (53.54), age (45.68), socioeconomic status (45.65), RBC transfusion (27.08), dementia (19.27), antiplatelet agents (16.81), gender (14.60), diabetes mellitus (13.00), liver disease (11.19), and PLT transfusion (10.11) (Figure 1). For example, the random forest variable importance of RBC transfusion for predicting all-cause mortality was 27. This means that, on average, the accuracy of the decision tree in the random forest will decrease by 0.05 if the RBC transfusion variable is removed from the model. Here, the number 0.05 was obtained by dividing 27 by 500 (the number of decision trees in the random forest). The top ten factors of the hospitalization period were CCI score, socioeconomic status, dementia, age, gender, hemiplegia, antiplatelet agents, diabetes mellitus, liver disease, and cardiovascular disease (Figure 2). The following supplementary tables are provided for additional reference: (1) the descriptive statistics of subgroups (Tables S2-1 to S2-16); and (2) the performance measures of machine learning for the prediction of all-cause mortality and hospitalization period (Tables S3-1 and S3-2).

## 4. Discussion

Transfusion requirements have been reported to be associated with poor clinical conditions [15]. A previous study reported that most patients with mild COVID-19 did not require blood transfusion, and RBC transfusion was necessary in severely ill patients, especially those with gastrointestinal bleeding, and the requirement for FFP and PLT transfusions was lower [18]. In contrast, another study reported that transfusion requirement was low, even in critically ill patients with COVID-19 [12]. In this study, blood transfusions were significantly associated with patients with COVID-19 with comorbidities. The percentage of patients with RBC transfusion was significantly higher in the high CCI score group than in the low CCI score group (5.5% vs. 0.3%). The trends for proportion of patients receiving PLT and FFP transfusions were comparable between the two groups (PLT, 1.5% vs. 0.1%; FFP, 1.0% vs. 0.1%). Moreover, blood transfusions have been reported as independent predictors of mortality in patients with COVID-19 [15,19]. A previous multivariable analysis of patients with COVID-19 showed that blood transfusions were associated with a significantly higher mortality rate [19]. The present study showed that patients who died during the study period received significantly more transfusions than those who survived (Table 4). Furthermore, when we conducted random forest analysis for correcting potential confounders, such as gender, socioeconomic status, comorbidities, and transfusion/medication history, RBC and PLT transfusions were the major determinants of mortality in patients with COVID-19 (Figure 1 and Figure 2). This finding was consistent with that of previous studies that analyzed blood transfusions and clinical outcomes in patients with COVID-19 [15,19]. 

Interestingly, in the random forest model, RBC and PLT transfusions were among the top ten factors of all-cause mortality. However, in a subgroup analysis based on CCI scores, only RBC transfusion maintained statistical significance for mortality in both the high (Group 1, Table S1-1) and low CCI score (Group 2, Table S1-2) groups. CCI score was the first-ranking factor for all-cause mortality in this study, and the validity of the CCI score for predicting mortality in patients with COVID-19 has been reported in previous studies [20,21,22]. Older age, low socioeconomic status, RBC transfusion, dementia, use of antiplatelet medications, gender (male), diabetes mellitus, liver disease, and PLT transfusion were also identified as important factors for all-cause mortality. This discrepancy between the two analyses might be because PLT transfusion has a statistically stronger interaction with CCI scores than with RBC transfusion, resulting in a bias. Thus, RBC transfusion can be a very strong factor for mortality in patients with COVID-19, because it maintained statistical significance even after correcting for bias using stratified subgroup analysis. This result is also concordant with that of a previous study that showed that the number of RBC units transfused is an independent factor for mortality in patients with COVID-19 [15]. Thus, restrictive RBC transfusion strategies might help to reduce the mortality in patients with COVID-19 independent of comorbidities. On the other hand, blood transfusion, even presumably appropriately administrated, depends on low values of hemoglobin. It is well known that anemia is a common feature of severe COVID-19 for many possible and combining reasons, including inflammation, marrow hypoproliferation, drugs, multiple blood sampling, red cell membrane damage, autoantibodies, hospitalization, and kidney damage [6,7,8,9]. RBC transfusion has also been used to mitigate low pulmonary perfusion [6,7,8,9]. Considering these aspects, it might be conceivable that more severe COVID-19 patients have transfused more than less severe patients. Therefore, further studies are needed to assess the associations of blood transfusions with the mortality and disease severity in patients with COVID-19.

We used machine learning and nationwide population data to analyze the associations of blood transfusion on all-cause mortality and hospitalization period in patients with COVID-19. Machine learning (or data mining) methods are statistical methods for “extracting knowledge from large amounts of data” [23]. Specifically, the random forest analysis does not require unrealistic assumptions of linear regression, such as ceteris paribus, “all the other variables remaining constant.” In addition, as demonstrated in this study, the random forest analysis can identify factors that are more important for the prediction of all-cause mortality and hospitalization period in patients with COVID-19. Further studies are needed, but this study will be a good starting point in this direction.

This study had some limitations. First, this study had the following methodological biases. As described above, this study used population data. Data of 7943 patients with COVID-19 diagnosed between 1 November 2019 and 31 May 2020 were obtained from the NHID. The NHID covers 52 million residents (nearly all residents) in Korea. All patients with any medical visit encoded as COVID-19 during the study period were included. The data size (7943) exceeds the minimum size to have desired properties with the 95% confidence interval and the 5% margin of error (156). However, the NHID provided limited information regarding the diagnosis, drugs, and service codes. Detailed information of individual patients was unavailable, including the dose of medication and laboratory data, such as hemoglobin level and PLT counts. Second, we used the ICD-10 codes to define the comorbidities included in this study. Thus, we would have underestimated the comorbidities of patients because the ICD-10 codes were broad and ambiguous. Finally, all patients included in this study were Korean. Therefore, our results should be applied cautiously to other populations.

## 5. Conclusions

In conclusion, this study based on a real-world population-based database showed that blood transfusions could be effective in patients with COVID-19. RBC and PLT transfusions were the major determinant factors for all-cause mortality based on machine learning analysis. In particular, a strong association between RBC transfusion and mortality was observed in patients with COVID-19. Thus, effective management of blood transfusion based on the comorbidities and disease severity in patients with COVID-19 may be beneficial. Further studies should evaluate the effect of blood transfusion in other populations with COVID-19.

## Figures and Tables

**Table 1:** Descriptive statistics (nominal variables) of patients with COVID-19 based on the Charlson Comorbidity Index score.

	ALL (n = 7943)			Group 1 (High Index, n = 3056)			Group 2 (Low Index, n = 4887)		
Variables	No (n)	Yes (n)	Yes (%)	No (n)	Yes (n)	Yes (%)	No (n)	Yes (n)	Yes (%)
Anemia	7422	521	6.56	2729	327	10.70	4693	194	3.97
Antiplatelet agents	6984	959	12.07	2177	879	28.76	4807	80	1.64
Congestive Heart Failure	7714	229	2.88	2856	200	6.54	4858	29	0.59
Chronic Kidney Disease	7872	71	0.89	2988	68	2.23	4884	3	0.06
Chronic Obstructive Pulmonary Disease	7289	654	8.23	2560	496	16.23	4729	158	3.23
Cryoprecipitate	7939	4	0.05	3053	3	0.10	4886	1	0.02
Connective Tissue Disease	7879	64	0.81	3004	52	1.70	4875	12	0.25
Cardiovascular Disease	7539	404	5.09	2672	384	12.57	4867	20	0.41
Death	7698	245	3.08	2815	241	7.89	4883	4	0.08
Dementia	7428	515	6.48	2549	507	16.59	4879	8	0.16
Diabetes Mellitus	6724	1219	15.35	2007	1049	34.33	4717	170	3.48
Fresh Frozen Plasma	7909	34	0.43	3027	29	0.95	4882	5	0.10
Hemiplegia	7853	90	1.13	2969	87	2.85	4884	3	0.06
Iron	7707	236	2.97	2894	162	5.30	4813	74	1.51
Leukemia	7937	6	0.08	3052	4	0.13	4885	2	0.04
Liver Disease	6549	1394	17.55	2056	1000	32.72	4493	394	8.06
Lymphoma	7934	9	0.11	3049	7	0.23	4885	2	0.04
Myocardial Infarction	7474	469	5.90	2693	363	11.88	4781	106	2.17
Platelet Transfusion	7891	52	0.65	3010	46	1.51	4881	6	0.12
Peptic Ulcer Disease	7109	834	10.50	2512	544	17.80	4597	290	5.93
Peripheral Vascular Disease	7539	404	5.09	2689	367	12.01	4850	37	0.76
Red Blood Cell Transfusion	7761	182	2.29	2888	168	5.50	4873	14	0.29
Gender	3177	4766	60.00	1183	1873	61.29	1994	2893	59.20
Solid Tumor	7656	287	3.61	2795	261	8.54	4861	26	0.53
Thrombocytopenia	7916	27	0.34	3037	19	0.62	4879	8	0.16
Tranexamic acid	7835	108	1.36	2970	86	2.81	4865	22	0.45

**Table 2:** Descriptive statistics (ordinal or continuous variables) of patients with COVID-19 based on the Charlson Comorbidity Index score.

All	Min	Q1	Median	Mean	Q3	Max
Age	0	2	4	4.17	6	8
Charlson Comorbidity Index	0	0	1	1.82	3	12
Hospitalization Period	2	21	33	48.67	53	329
Insurance Fee	0	3	10	10.00	16	20
**Group 1 (High Index)**						
Age	3	5	6	6.16	7	8
Charlson Comorbidity Index	2	3	4	4.07	5	12
Hospitalization Period	2	33	52	74.52	90	329
Insurance Fee	0	2	10	9.62	16	20
**Group 2 (Low Index)**						
Age	0	2	3	2.93	4	5
Charlson Comorbidity Index	0	0	0	0.42	1	3
Hospitalization Period	2	19	27	32.51	39	270
Insurance Fee	0	4	11	10.24	16	20

**Table 3:** Blood transfusion vs. Charlson Comorbidity Index score.

			No.	Proportion (%)
All	Group 1 (High Index)		3056	38.5
	Group 2 (Low Index)		4887	61.5
Red Blood Cell	Group 1	Non-transfused	2888	94.5
		Transfused	168	5.5
	Group 2	Non-transfused	4873	99.7
		Transfused	14	0.3
Platelet	Group 1	Non-transfused	3010	98.5
		Transfused	46	1.5
	Group 2	Non-transfused	4881	99.9
		Transfused	6	0.1
Fresh Frozen Plasma	Group 1	Non-transfused	3027	99
		Transfused	29	1
	Group 2	Non-transfused	4882	99.9
		Transfused	5	0.1
Cryoprecipitate	Group 1	Non-transfused	3053	99.9
		Transfused	3	0.1
	Group 2	Non-transfused	4886	99.9
		Transfused	1	0.1

Note: the proportions of red blood cell, platelet, and fresh frozen plasma transfusions were significantly higher in Group 1 (high CCI scores) than in Group 2 (low CCI scores) (*p* < 0.01 for the *t* test).

**Table 4:** Blood transfusion vs. all-cause mortality.

			No.	Proportion (%)
All	Dead		980	3.1
	Alive		30791	96.9
Red Blood Cell	Dead	Non-transfused	172	70.2
		Transfused	73	29.8
	Alive	Non-transfused	7589	98.6
		Transfused	109	1.4
Platelet	Dead	Non-transfused	217	11.4
		Transfused	28	88.6
	Alive	Non-transfused	7674	99.7
		Transfused	24	0.3
Fresh Frozen Plasma	Dead	Non-transfused	228	93.1
		Transfused	17	6.9
	Alive	Non-transfused	7681	99.8
		Transfused	17	0.2
Cryoprecipitate	Dead	Non-transfused	243	99.2
		Transfused	2	0.8
	Alive	Non-transfused	7696	99.9
		Transfused	2	0.1

Note: the proportions of red blood cell, platelet, fresh frozen plasma, and cryoprecipitate transfusions were significantly higher in patients who died than in survivors (*p* < 0.01).

## Data Availability

The data presented in this study are not publicly available. However, the data are available from the corresponding author upon reasonable request and under the permission of Korea National Health Insurance Service.

## Supplementary Materials

The following supporting information can be downloaded at: https://www.mdpi.com/article/10.3390/diagnostics12122970/s1, Table S1-1. Blood Transfusion vs. All-Cause Mortality: G1 (High Index); Table S1-2. Blood Transfusion vs. All-Cause Mortality: G2 (Low Index); Table S2-1. Descriptive Statistics of Group1 without Red-Cell Transfusion (2888); Table S2-2. Descriptive Statistics of Group 1 with Red-Cell Transfusion (168); Table S2-3. Descriptive Statistics of Group 2 without Red-Cell Transfusion (4873); Table S2-4. Descriptive Statistics of Group 2 with Red-Cell Transfusion (14); Table S2-5. Descriptive Statistics of Group 1 without Platelet Transfusion (3010); Table S2-6. De-scriptive Statistics of Group 1 with Platelet Transfusion (46); Table S2-7. Descriptive Statistics of Group 2 without Platelet Transfusion (4881); Table S2-8. Descriptive Statistics of Group 2 with Platelet Transfusion (6); Table S2-9. Descriptive Statistics of Group 1 without Fresh Frozen Plasma Transfusion (3027); Table S2-10. Descriptive Statistics of Group 1 with Fresh Frozen Plasma Transfusion (29); Table S2-11. Descriptive Statistics of Group 2 without Fresh Frozen Plasma Transfusion (4882); Table S2-12. Descriptive Statistics of Group 2 with Fresh Frozen Plasma Transfusion (5); Table S2-13. Descriptive Statistics of Group 1 without Cryoprecipitate Transfusion (3053); Table S2-14. Descriptive Statistics of Group 1 with Cryoprecipitate Transfusion (3); Table S2-15. Descriptive Statistics of Group 2 without Cryoprecipitate Transfusion (4886); Table S2-16. Descriptive Statistics of Group 2 with Cryoprecipitate Transfusion (1); Table S3-1. Model Perfor-mance for Death; Table S3-2. Model Performance for Hospitalization Period.

## References

[1] Sharma A., Tiwari S., Deb M.K., Marty J.L. (2020). Severe acute respiratory syndrome coronavirus-2 (SARS-CoV-2): A global pandemic and treatment strategies. Int. J. Antimicrob. Agents.

[2] Chauhan S. (2020). Comprehensive review of coronavirus disease 2019 (COVID-19). Biomed. J..

[3] Lai C.C., Shih T.P., Ko W.C., Tang H.J., Hsueh P.R. (2020). Severe acute respiratory syndrome coronavirus 2 (SARS-CoV-2) and coronavirus disease-2019 (COVID-19): The epidemic and the challenges. Int. J. Antimicrob. Agents.

[4] Rapp J.L., Lieberman-Cribbin W., Tuminello S., Taioli E. (2021). Male sex, severe obesity, older age, and chronic kidney disease are associated with COVID-19 severity and mortality in New York City. Chest.

[5] Poly T.N., Islam M.M., Yang H.C., Lin M.C., Jian W.-S., Hsu M.-H., Li Y.-C.J. (2021). Obesity and mortality among patients diagnosed with COVID-19: A systematic review and meta-analysis. Front. Med..

[6] Bellmann-Weiler R., Lanser L., Barket R., Rangger L., Schapfl A., Schaber M., Fritsche G., Wöll E., Weiss G. (2020). Prevalence and predictive value of anemia and dysregulated iron homeostasis in patients with COVID-19 infection. J. Clin. Med..

[7] Faghih D.M., Somi M.H., Sadeghi M.E., Abbasalizad F.M., Nikniaz Z. (2021). Anemia predicts poor outcomes of COVID-19 in hospitalized patients: A prospective study in Iran. BMC Infect. Dis..

[8] Oh S.M., Skendelas J.P., Macdonald E., Bergamini M., Goel S., Choi J., Segal K.R., Vivek K., Nair S., Leff J. (2021). On-admission anemia predicts mortality in COVID-19 patients: A single center, retrospective cohort study. Am. J. Emerg. Med..

[9] Tao Z., Xu J., Chen W., Yang Z., Xu X., Liu L., Chen R., Xie J., Liu M., Wu J. (2021). Anemia is associated with severe illness in COVID-19: A retrospective cohort study. J. Med. Virol..

[10] Young B.E., Ong S.W.X., Kalimuddin S., Low J.G., Tan S.Y., Loh J., Ng O.T., Marimuthu K., Ang L.W., Mak T.M. (2020). Epidemiologic features and clinical course of patients infected with SARS-CoV-2 in Singapore. JAMA.

[11] Cecconi M., Piovani D., Brunetta E., Aghemo A., Greco M., Ciccarelli M., Angelini C., Voza A., Omodei P., Vespa E. (2020). Early predictors of clinical deterioration in a cohort of 239 patients hospitalized for COVID-19 infection in Lombardy, Italy. J. Clin. Med..

[12] Stanworth S.J., New H.V., Apelseth T.O., Brunskill S., Cardigan R., Doree C., Germain M., Goldman M., Massey E., Prati D. (2020). Effects of the COVID-19 pandemic on supply and use of blood for transfusion. Lancet Haematol..

[13] Al Mahmasani L., Hodroj M.H., Finianos A., Taher A. (2021). COVID-19 pandemic and transfusion medicine: The worldwide challenge and its implications. Ann. Hematol..

[14] Barriteau C.M., Bochey P., Lindholm P.F., Hartman K., Sumugod R., Ramsey G. (2020). Blood transfusion utilization in hospitalized COVID-19 patients. Transfusion..

[15] Grandone E., Pesavento R., Tiscia G., De Laurenzo A., Ceccato D., Sartori M., Mirabella L., Cinnella G., Mastroianno M., Dalfino L. (2021). Mortality and transfusion requirements in COVID-19 hospitalized Italian patients according to severity of the disease. J. Clin. Med..

[16] Charlson M.E., Pompei P., Ales K.L., MacKenzie C.R. (1987). A new method of classifying prognostic comorbidity in longitudinal studies: Development and validation. J. Chronic Dis..

[17] Myttenaere A.D., Golden B., Grand B.L., Rossi F. (2016). Mean Absolute Percentage Error for regression models. Neurocomputing.

[18] Fan B.E., Ong K.H., Chan S.S.W., Young B.E., Chong V.C.L., Chen S.P.C., Lim S.P., Lim G.P., Kuperan P. (2020). Blood and blood product use during COVID-19 infection. Am. J. Hematol..

[19] Rim J.H., Lee S.A., Han C.H., Yoo J. (2020). Transfusion demand in COVID-19 patients from the Korean population: A nationwide study in South Korea. Br. J. Haematol..

[20] Cho S.I., Yoon S., Lee H.J. (2021). Impact of comorbidity burden on mortality in patients with COVID-19 using the Korean health insurance database. Sci. Rep..

[21] Tuty Kuswardhani R.A., Henrina J., Pranata R., Anthonius L.M., Lawrensia S., Suastika K. (2020). Charlson comorbidity index and a composite of poor outcomes in COVID-19 patients: A systematic review and meta-analysis. Diabetes Metab. Syndr..

[22] Varol Y., Hakoglu B., Kadri Cirak A., Polat G., Komurcuoglu B., Akkol B., Atasoy C., Bayramic E., Balci G., Ataman S. (2021). The impact of Charlson comorbidity index on mortality from SARS-CoV-2 virus infection and A novel COVID-19 mortality index: CoLACD. Int. J. Clin. Pract..

[23] Han J., Micheline K. (2006). Data Mining: Concepts and Techniques.

