# Supplementary material for: Blood Transfusion, All-Cause Mortality and Hospitalization Period in COVID-19 Patients: Machine Learning Analysis of National Health Insurance Claims Data
Source: Diagnostics (Basel). 2022 Nov 28;12(12):2970. doi: 10.3390/diagnostics12122970 (PMC9777003; doi:10.3390/diagnostics12122970)
Supplement: Supplementary file 1 [file diagnostics-12-02970-s001.zip › diagnostics-2020842-supplementary.pdf]

Table S1-1. Blood Transfusion vs. All-Cause Mortality: G1 (High Index)

			Count	Proportion (%)
G1 All	Death Yes		241	7.9
	Death No		2815	92.1
Red Cell	Death Yes	X	170	70.5
		O	71	29.5
	Death No	X	2718	96.6
		O	97	3.4
Platelet	Death Yes	X	214	88.8
		O	27	11.2
	Death No	X	2796	99.3
		O	19	0.7
Fresh Frozen Plasma	Death Yes	X	225	93.4
		O	16	6.6
	Death No	X	2802	99.5
		O	13	0.5
Cryoprecipitate	Death Yes	X	239	99.2
		O	2	0.8
	Death No	X	2814	100.0
		O	1	0.0

Note: The proportion of red-cell transfusion was significantly higher in those with death than in those without death, i.e., P-values < 0.01 for the t test

Table S1-2. Blood Transfusion vs. All-Cause Mortality: G2 (Low Index)

			Count	Proportion (%)
G2 All	Death Yes		4	0.1
	Death No		4883	99.9
Red Cell	Death Yes	X	2	50.0
		O	2	50.0
	Death No	X	4871	99.8
		O	12	0.2
Platelet	Death Yes	X	3	75.0
		O	1	25.0
	Death No	X	4878	99.9
		O	5	0.1
Fresh Frozen Plasma	Death Yes	X	3	75.0
		O	1	25.0
	Death No	X	4879	99.9
		O	4	0.1
Cryoprecipitate	Death Yes	X	4	100.0
		O	0	0.0
	Death No	X	4882	100.0
		O	1	0.0

Note: The proportion of red-cell transfusion was significantly higher in those with death than in those without death, i.e., P-values < 0.05 for the t test.

Table S2-1. Descriptive Statistics of Group1 without Red-Cell Transfusion (2888)

**Variable**	*No*	*Yes*	*Yes (%)*
Anemia	2626	262	9.07
Anti-platelet	2143	745	25.80
Congestive Heart Failure	2712	176	6.09
Chronic Kidney Disease	2846	42	1.45
Chronic Obstructive Pulmonary Disease	2433	455	15.75
Cryoprecipitate	2888	0	0.00
Connective Tissue Disease	2841	47	1.63
Cardiovascular Disease	2539	349	12.08
Death	2718	170	5.89
Dementia	2439	449	15.55
Diabetes Mellitus	1917	971	33.62
Fresh Frozen Plasma	2886	2	0.07
Hemiplegia	2810	78	2.70
Iron	2785	103	3.57
Leukemia	2885	3	0.10
Liver Disease	1934	954	33.03
Lymphoma	2882	6	0.21
Myocardial Infarction	2553	335	11.60
Platelets	2884	4	0.14
Peptic Ulcer Disease	2380	508	17.59
Peripheral Vascular Disease	2549	339	11.74
Red Cell	2888	0	0.00
Sex	1102	1786	61.84
Solid Tumor	2658	230	7.96
Thrombocytopenia	2879	9	0.31
Tranexamic	2839	49	1.70

	**Min**	**Q1**	**Median**	**Mean**	**Q3**	**Max**
Age	3	5	6	6.12	7	8
Charlson Comorbidity Index	2	2	4	3.96	5	12
Hospitalization Period	2	32	50	71.86	85	329

Table S2-2. Descriptive Statistics of Group 1 with Red-Cell Transfusion (168)

**Variable**	*No*	*Yes*	*Yes (%)*
Anemia	103	65	38.69
Anti-platelet	34	134	79.76
Congestive Heart Failure	144	24	14.29
Chronic Kidney Disease	142	26	15.48
Chronic Obstructive Pulmonary Disease	127	41	24.40
Cryoprecipitate	165	3	1.79
Connective Tissue Disease	163	5	2.98
Cardiovascular Disease	133	35	20.83
Death	97	71	42.26
Dementia	110	58	34.52
Diabetes Mellitus	90	78	46.43
Fresh Frozen Plasma	141	27	16.07
Hemiplegia	159	9	5.36
Iron	109	59	35.12
Leukemia	167	1	0.60
Liver Disease	122	46	27.38
Lymphoma	167	1	0.60
Myocardial Infarction	140	28	16.67
Platelets	126	42	25.00
Peptic Ulcer Disease	132	36	21.43
Peripheral Vascular Disease	140	28	16.67
Red Cell	0	168	100.00
Sex	81	87	51.79
Solid Tumor	137	31	18.45
Thrombocytopenia	158	10	5.95
Tranexamic	131	37	22.02

	**Min**	**Q1**	**Median**	**Mean**	**Q3**	**Max**
Age	4	6	7	6.90	8	8
Charlson Comorbidity Index	2	5	6	5.96	7	12
Hospitalization Period	18	63	102.5	120.30	164.8	313

Table S2-3. Descriptive Statistics of Group 2 without Red-Cell Transfusion (4873)

**Variable**	*No*	*Yes*	*Yes (%)*
Anemia	4685	188	3.86
Anti-platelet	4801	72	1.48
Congestive Heart Failure	4845	28	0.57
Chronic Kidney Disease	4870	3	0.06
Chronic Obstructive Pulmonary Disease	4715	158	3.24
Cryoprecipitate	4873	0	0.00
Connective Tissue Disease	4862	11	0.23
Cardiovascular Disease	4853	20	0.41
Death	4871	2	0.04
Dementia	4866	7	0.14
Diabetes Mellitus	4705	168	3.45
Fresh Frozen Plasma	4872	1	0.02
Hemiplegia	4870	3	0.06
Iron	4804	69	1.42
Leukemia	4871	2	0.04
Liver Disease	4482	391	8.02
Lymphoma	4871	2	0.04
Myocardial Infarction	4767	106	2.18
Platelets	4870	3	0.06
Peptic Ulcer Disease	4583	290	5.95
Peripheral Vascular Disease	4836	37	0.76
Red Cell	4873	0	0.00
Sex	1987	2886	59.22
Solid Tumor	4847	26	0.53
Thrombocytopenia	4865	8	0.16
Tranexamic	4852	21	0.43

	**Min**	**Q1**	**Median**	**Mean**	**Q3**	**Max**
Age	0	2	3	2.92	4	5
Charlson Comorbidity Index	0	0	0	0.41	1	3
Hospitalization Period	2	19	27	32.40	38	270

Table S2-4. Descriptive Statistics of Group 2 with Red-Cell Transfusion (14)

**Variable**	*No*	*Yes*	*Yes (%)*
Anemia	8	6	42.86
Anti-platelet	6	8	57.14
Congestive Heart Failure	13	1	7.14
Chronic Kidney Disease	14	0	0.00
Chronic Obstructive Pulmonary Disease	14	0	0.00
Cryoprecipitate	13	1	7.14
Connective Tissue Disease	13	1	7.14
Cardiovascular Disease	14	0	0.00
Death	12	2	14.29
Dementia	13	1	7.14
Diabetes Mellitus	12	2	14.29
Fresh Frozen Plasma	10	4	28.57
Hemiplegia	14	0	0.00
Iron	9	5	35.71
Leukemia	14	0	0.00
Liver Disease	11	3	21.43
Lymphoma	14	0	0.00
Myocardial Infarction	14	0	0.00
Platelets	11	3	21.43
Peptic Ulcer Disease	14	0	0.00
Peripheral Vascular Disease	14	0	0.00
Red Cell	0	14	100.00
Sex	7	7	50.00
Solid Tumor	14	0	0.00
Thrombocytopenia	14	0	0.00
Tranexamic	13	1	7.14

	**Min**	**Q1**	**Median**	**Mean**	**Q3**	**Max**
Age	1	3	4	3.64	4	5
Charlson Comorbidity Index	0	0	1	0.79	1	2
Hospitalization Period	3	33.25	60	68.29	85.25	209

Table S2-5. Descriptive Statistics of Group 1 without Platelet Transfusion (3010)

**Variable**	*No*	*Yes*	*Yes (%)*
Anemia	2697	313	10.40
Anti-platelet	2174	836	27.77
Congestive Heart Failure	2820	190	6.31
Chronic Kidney Disease	2952	58	1.93
Chronic Obstructive Pulmonary Disease	2524	486	16.15
Cryoprecipitate	3010	0	0.00
Connective Tissue Disease	2960	50	1.66
Cardiovascular Disease	2638	372	12.36
Death	2796	214	7.11
Dementia	2520	490	16.28
Diabetes Mellitus	1986	1024	34.02
Fresh Frozen Plasma	3006	4	0.13
Hemiplegia	2925	85	2.82
Iron	2863	147	4.88
Leukemia	3007	3	0.10
Liver Disease	2023	987	32.79
Lymphoma	3003	7	0.23
Myocardial Infarction	2658	352	11.69
Platelets	3010	0	0.00
Peptic Ulcer Disease	2475	535	17.77
Peripheral Vascular Disease	2650	360	11.96
Red Cell	2884	126	4.19
Sex	1153	1857	61.69
Solid Tumor	2756	254	8.44
Thrombocytopenia	3001	9	0.30
Tranexamic	2939	71	2.36

	**Min**	**Q1**	**Median**	**Mean**	**Q3**	**Max**
Age	3	5	6	6.15	7	8
Charlson Comorbidity Index	2	2	4	4.04	5	12
Hospitalization Period	2	32	51	73.87	90	329

Table S2-6. Descriptive Statistics of Group 1 with Platelet Transfusion (46)

**Variable**	*No*	*Yes*	*Yes (%)*
Anemia	32	14	30.43
Anti-platelet	3	43	93.48
Congestive Heart	36	10	21.74
Chronic Kidney	36	10	21.74
Chronic Obstructive	36	10	21.74
Cryoprecipitate	43	3	6.52
Connective Tissue	44	2	4.35
Cardiovascular Disease	34	12	26.09
Death	19	27	58.70
Dementia	29	17	36.96
Diabetes Mellitus	21	25	54.35
Fresh Frozen Plasma	21	25	54.35
Hemiplegia	44	2	4.35
Iron	31	15	32.61
Leukemia	45	1	2.17
Liver Disease	33	13	28.26
Lymphoma	46	0	0.00
Myocardial Infarction	35	11	23.91
Platelets	0	46	100.00
Peptic Ulcer Disease	37	9	19.57
Peripheral Vascular	39	7	15.22
Red Cell	4	42	91.30
Sex	30	16	34.78
Solid Tumor	39	7	15.22
Thrombocytopenia	36	10	21.74
Tranexamic	31	15	32.61

	**Min**	**Q1**	**Median**	**Mean**	**Q3**	**Max**
Age	5	6	7	6.65	7.75	8
Charlson Comorbidity Index	2	5	6	6.04	8	10
Hospitalization Period	23	63	89.5	117.4	153.5	260

Table S2-7. Descriptive Statistics of Group 2 without Platelet Transfusion (4881)

**Variable**	*No*	*Yes*	*Yes (%)*
Anemia	4687	194	3.97
Anti-platelet	4805	76	1.56
Congestive Heart Failure	4854	27	0.55
Chronic Kidney Disease	4878	3	0.06
Chronic Obstructive Pulmonary Disease	4724	157	3.22
Cryoprecipitate	4880	1	0.02
Connective Tissue Disease	4869	12	0.25
Cardiovascular Disease	4861	20	0.41
Death	4878	3	0.06
Dementia	4874	7	0.14
Diabetes Mellitus	4712	169	3.46
Fresh Frozen Plasma	4878	3	0.06
Hemiplegia	4878	3	0.06
Iron	4807	74	1.52
Leukemia	4879	2	0.04
Liver Disease	4488	393	8.05
Lymphoma	4879	2	0.04
Myocardial Infarction	4775	106	2.17
Platelets	4881	0	0.00
Peptic Ulcer Disease	4591	290	5.94
Peripheral Vascular Disease	4844	37	0.76
Red Cell	4870	11	0.23
Sex	1989	2892	59.25
Solid Tumor	4855	26	0.53
Thrombocytopenia	4875	6	0.12
Tranexamic	4859	22	0.45

	**Min**	**Q1**	**Median**	**Mean**	**Q3**	**Max**
Age	0	2	3	2.93	4	5
Charlson Comorbidity Index	0	0	0	0.41	1	3
Hospitalization Period	2	19	27	32.43	39	270

Table S2-8. Descriptive Statistics of Group 2 with Platelet Transfusion (6)

**Variable**	*No*	*Yes*	*Yes (%)*
Anemia	6	0	0.00
Anti-platelet	2	4	66.67
Congestive Heart Failure	4	2	33.33
Chronic Kidney Disease	6	0	0.00
Chronic Obstructive Pulmonary Disease	5	1	16.67
Cryoprecipitate	6	0	0.00
Connective Tissue Disease	6	0	0.00
Cardiovascular Disease	6	0	0.00
Death	5	1	16.67
Dementia	5	1	16.67
Diabetes Mellitus	5	1	16.67
Fresh Frozen Plasma	4	2	33.33
Hemiplegia	6	0	0.00
Iron	6	0	0.00
Leukemia	6	0	0.00
Liver Disease	5	1	16.67
Lymphoma	6	0	0.00
Myocardial Infarction	6	0	0.00
Platelets	0	6	100.00
Peptic Ulcer Disease	6	0	0.00
Peripheral Vascular Disease	6	0	0.00
Red Cell	3	3	50.00
Sex	5	1	16.67
Solid Tumor	6	0	0.00
Thrombocytopenia	4	2	33.33
Tranexamic	6	0	0.00

	**Min**	**Q1**	**Median**	**Mean**	**Q3**	**Max**
Age	2	4	4	4.00	4.75	5
Charlson Comorbidity Index	1	1	1	1.33	1.75	2
Hospitalization Period	31	41.75	57.50	95.00	104.75	267

Table S2-9. Descriptive Statistics of Group 1 without Fresh Frozen Plasma Transfusion (3027)

**Variable**	*No*	*Yes*	*Yes (%)*
Anemia	2707	320	10.57
Anti-platelet	2176	851	28.11
Congestive Heart Failure	2833	194	6.41
Chronic Kidney Disease	2964	63	2.08
Chronic Obstructive Pulmonary Disease	2539	488	16.12
Cryoprecipitate	3027	0	0.00
Connective Tissue Disease	2976	51	1.68
Cardiovascular Disease	2649	378	12.49
Death	2802	225	7.43
Dementia	2529	498	16.45
Diabetes Mellitus	1995	1032	34.09
Fresh Frozen Plasma	3027	0	0.00
Hemiplegia	2940	87	2.87
Iron	2873	154	5.09
Leukemia	3024	3	0.10
Liver Disease	2036	991	32.74
Lymphoma	3020	7	0.23
Myocardial Infarction	2671	356	11.76
Platelets	3006	21	0.69
Peptic Ulcer Disease	2490	537	17.74
Peripheral Vascular Disease	2665	362	11.96
Red Cell	2886	141	4.66
Sex	1164	1863	61.55
Solid Tumor	2771	256	8.46
Thrombocytopenia	3013	14	0.46
Tranexamic	2953	74	2.44

	**Min**	**Q1**	**Median**	**Mean**	**Q3**	**Max**
Age	3	5	6	6.16	7	8
Charlson Comorbidity Index	2	3	4	4.05	5	12
Hospitalization Period	2	32	51	74.16	90	329

Table S2-10. Descriptive Statistics of Group 1 with Fresh Frozen Plasma Transfusion (29)

**Variable**	*No*	*Yes*	*Yes (%)*
Anemia	22	7	24.14
Anti-platelet	1	28	96.55
Congestive Heart Failure	23	6	20.69
Chronic Kidney Disease	24	5	17.24
Chronic Obstructive Pulmonary Disease	21	8	27.59
Cryoprecipitate	26	3	10.34
Connective Tissue Disease	28	1	3.45
Cardiovascular Disease	23	6	20.69
Death	13	16	55.17
Dementia	20	9	31.03
Diabetes Mellitus	12	17	58.62
Fresh Frozen Plasma	0	29	100.00
Hemiplegia	29	0	0.00
Iron	21	8	27.59
Leukemia	28	1	3.45
Liver Disease	20	9	31.03
Lymphoma	29	0	0.00
Myocardial Infarction	22	7	24.14
Platelets	4	25	86.21
Peptic Ulcer Disease	22	7	24.14
Peripheral Vascular Disease	24	5	17.24
Red Cell	2	27	93.10
Sex	19	10	34.48
Solid Tumor	24	5	17.24
Thrombocytopenia	24	5	17.24
Tranexamic	17	12	41.38

	**Min**	**Q1**	**Median**	**Mean**	**Q3**	**Max**
Age	5	6	6	6.35	7	8
Charlson Comorbidity Index	2	4	6	5.69	7	10
Hospitalization Period	38	63	107	112.40	137	313

Table S2-11. Descriptive Statistics of Group 2 without Fresh Frozen Plasma Transfusion (4882)

**Variable**	*No*	*Yes*	*Yes (%)*
Anemia	4688	194	3.97
Anti-platelet	4807	75	1.54
Congestive Heart Failure	4853	29	0.59
Chronic Kidney Disease	4879	3	0.06
Chronic Obstructive Pulmonary Disease	4724	158	3.24
Cryoprecipitate	4882	0	0.00
Connective Tissue Disease	4870	12	0.25
Cardiovascular Disease	4862	20	0.41
Death	4879	3	0.06
Dementia	4875	7	0.14
Diabetes Mellitus	4712	170	3.48
Fresh Frozen Plasma	4882	0	0.00
Hemiplegia	4879	3	0.06
Iron	4808	74	1.52
Leukemia	4880	2	0.04
Liver Disease	4490	392	8.03
Lymphoma	4880	2	0.04
Myocardial Infarction	4776	106	2.17
Platelets	4878	4	0.08
Peptic Ulcer Disease	4592	290	5.94
Peripheral Vascular Disease	4845	37	0.76
Red Cell	4872	10	0.20
Sex	1991	2891	59.22
Solid Tumor	4856	26	0.53
Thrombocytopenia	4875	7	0.14
Tranexamic	4861	21	0.43

	**Min**	**Q1**	**Median**	**Mean**	**Q3**	**Max**
Age	0	2	3	2.93	4	5
Charlson Comorbidity Index	0	0	0	0.41	1	3
Hospitalization Period	2	19	27	32.47	39	270

Table S2-12. Descriptive Statistics of Group 2 with Fresh Frozen Plasma Transfusion (5)

**Variable**	*No*	*Yes*	*Yes (%)*
Anemia	5	0	0.00
Anti-platelet	0	5	100.00
Congestive Heart Failure	5	0	0.00
Chronic Kidney Disease	5	0	0.00
Chronic Obstructive Pulmonary Disease	5	0	0.00
Cryoprecipitate	4	1	20.00
Connective Tissue Disease	5	0	0.00
Cardiovascular Disease	5	0	0.00
Death	4	1	20.00
Dementia	4	1	20.00
Diabetes Mellitus	5	0	0.00
Fresh Frozen Plasma	0	5	100.00
Hemiplegia	5	0	0.00
Iron	5	0	0.00
Leukemia	5	0	0.00
Liver Disease	3	2	40.00
Lymphoma	5	0	0.00
Myocardial Infarction	5	0	0.00
Platelets	3	2	40.00
Peptic Ulcer Disease	5	0	0.00
Peripheral Vascular Disease	5	0	0.00
Red Cell	1	4	80.00
Sex	3	2	40.00
Solid Tumor	5	0	0.00
Thrombocytopenia	4	1	20.00
Tranexamic	4	1	20.00

	**Min**	**Q1**	**Median**	**Mean**	**Q3**	**Max**
Age	3	4	5	4.40	5	5
Charlson Comorbidity Index	1	1	1	1.20	1	2
Hospitalization Period	3	41	90	72.20	111	116

Table S2-13. Descriptive Statistics of Group 1 without Cryoprecipitate Transfusion (3053)

**Variable**	*No*	*Yes*	*Yes (%)*
Anemia	2726	327	10.71
Anti-platelet	2177	876	28.69
Congestive Heart Failure	2855	198	6.49
Chronic Kidney Disease	2985	68	2.23
Chronic Obstructive Pulmonary Disease	2558	495	16.21
Cryoprecipitate	3053	0	0.00
Connective Tissue Disease	3001	52	1.70
Cardiovascular Disease	2670	383	12.55
Death	2814	239	7.83
Dementia	2546	507	16.61
Diabetes Mellitus	2006	1047	34.29
Fresh Frozen Plasma	3027	26	0.85
Hemiplegia	2966	87	2.85
Iron	2893	160	5.24
Leukemia	3049	4	0.13
Liver Disease	2054	999	32.72
Lymphoma	3046	7	0.23
Myocardial Infarction	2692	361	11.82
Platelets	3010	43	1.41
Peptic Ulcer Disease	2510	543	17.79
Peripheral Vascular Disease	2687	366	11.99
Red Cell	2888	165	5.40
Sex	1183	1870	61.25
Solid Tumor	2792	261	8.55
Thrombocytopenia	3034	19	0.62
Tranexamic	2968	85	2.78

	**Min**	**Q1**	**Median**	**Mean**	**Q3**	**Max**
Age	5	5	6	6.16	7	8
Charlson Comorbidity Index	2	3	4	4.06	5	12
Hospitalization Period	2	33	52	74.51	90	329

Table S2-14. Descriptive Statistics of Group 1 with Cryoprecipitate Transfusion (3)

**Variable**	*No*	*Yes*	*Yes (%)*
Anemia	3	0	0.00
Anti-platelet	0	3	100.00
Congestive Heart Failure	1	2	66.67
Chronic Kidney Disease	3	0	0.00
Chronic Obstructive Pulmonary Disease	2	1	33.33
Cryoprecipitate	0	3	100.00
Connective Tissue Disease	3	0	0.00
Cardiovascular Disease	2	1	33.33
Death	1	2	66.67
Dementia	3	0	0.00
Diabetes Mellitus	1	2	66.67
Fresh Frozen Plasma	0	3	100.00
Hemiplegia	3	0	0.00
Iron	1	2	66.67
Leukemia	3	0	0.00
Liver Disease	2	1	33.33
Lymphoma	3	0	0.00
Myocardial Infarction	1	2	66.67
Platelets	0	3	100.00
Peptic Ulcer Disease	2	1	33.33
Peripheral Vascular Disease	2	1	33.33
Red Cell	0	3	100.00
Sex	0	3	100.00
Solid Tumor	3	0	0.00
Thrombocytopenia	3	0	0.00
Tranexamic	2	1	33.33

	**Min**	**Q1**	**Median**	**Mean**	**Q3**	**Max**
Age	6	6.5	7	6.67	7	7
Charlson Comorbidity Index	4	5.5	7	6.33	7.5	8
Hospitalization Period	61	61	61	82.67	93.5	126

Table S2-15. Descriptive Statistics of Group 2 without Cryoprecipitate Transfusion (4886)

**Variable**	*No*	*Yes*	*Yes (%)*
Anemia	4692	194	3.97
Anti-platelet	4807	79	1.62
Congestive Heart Failure	4857	29	0.59
Chronic Kidney Disease	4883	3	0.06
Chronic Obstructive Pulmonary Disease	4728	158	3.23
Cryoprecipitate	4886	0	0.00
Connective Tissue Disease	4874	12	0.25
Cardiovascular Disease	4866	20	0.41
Death	4882	4	0.08
Dementia	4878	8	0.16
Diabetes Mellitus	4716	170	3.48
Fresh Frozen Plasma	4882	4	0.08
Hemiplegia	4883	3	0.06
Iron	4812	74	1.51
Leukemia	4884	2	0.04
Liver Disease	4492	394	8.06
Lymphoma	4884	2	0.04
Myocardial Infarction	4780	106	2.17
Platelets	4880	6	0.12
Peptic Ulcer Disease	4596	290	5.94
Peripheral Vascular Disease	4849	37	0.76
Red Cell	4873	13	0.27
Sex	1994	2892	59.19
Solid Tumor	4860	26	0.53
Thrombocytopenia	4878	8	0.16
Tranexamic	4864	22	0.45

	**Min**	**Q1**	**Median**	**Mean**	**Q3**	**Max**
Age	0	2	3	2.93	4	5
Charlson Comorbidity Index	0	0	0	0.42	1	3
Hospitalization Period	2	19	27	32.49	39	270

Table S2-16. Descriptive Statistics of Group 2 with Cryoprecipitate Transfusion (1)

**Variable**	*No*	*Yes*	*Yes (%)*
Anemia	1	0	0.00
Anti-platelet	0	1	100.00
Congestive Heart Failure	1	0	0.00
Chronic Kidney Disease	1	0	0.00
Chronic Obstructive Pulmonary Disease	1	0	0.00
Cryoprecipitate	0	1	100.00
Connective Tissue Disease	1	0	0.00
Cardiovascular Disease	1	0	0.00
Death	1	0	0.00
Dementia	1	0	0.00
Diabetes Mellitus	1	0	0.00
Fresh Frozen Plasma	0	1	100.00
Hemiplegia	1	0	0.00
Iron	1	0	0.00
Leukemia	1	0	0.00
Liver Disease	1	0	0.00
Lymphoma	1	0	0.00
Myocardial Infarction	1	0	0.00
Platelets	1	0	0.00
Peptic Ulcer Disease	1	0	0.00
Peripheral Vascular Disease	1	0	0.00
Red Cell	0	1	100.00
Sex	0	1	100.00
Solid Tumor	1	0	0.00
Thrombocytopenia	1	0	0.00
Tranexamic	1	0	0.00

	**Min**	**Q1**	**Median**	**Mean**	**Q3**	**Max**
Age	5	5	5	5	5	5
Charlson Comorbidity Index	1	1	1	1	1	1
Hospitalization Period	111	111	111	111	111	111

Table S3-1. Model Performance for Death

			Random Forest		Logistic Regression	
			Precision	AUC	Precision	AUC
Red Cell	G1	X	0.9453	0.8198	0.9373	0.8586
		O	0.6275	0.6417	0.5294	0.4798
	G2	X	0.9992	0.5000	1.0000	-
		O	0.7500	0.6667	0.7500	0.3333
Platelet	G1	X	0.9347	0.8386	0.9395	0.8568
		O	0.3333	0.3864	0.5333	0.5000
	G2	X	0.9992	0.4970	1.0000	-
		O	0.5000	1.0000	0.0000	-
Fresh Frozen Plasma	G1	X	0.9331	0.8666	0.9302	0.8872
		O	0.6000	0.7083	0.8000	0.8000
	G2	X	1.0000	-	0.9992	0.3183
		O	1.0000	-	0.0000	-
Cryoprecipitate	G1	X	0.9355	0.8800	0.9298	0.8816
		O	-	-	-	-
	G2	X	0.9985	1.0000	0.9992	0.3127
		O	-	-	-	-

Table S3-2. Model Performance for Hospitalization Period

			Random Forest		Linear Regression	
			R-Square	MAPE	R-Square	MAPE
Red Cell	G1	X	0.2870	0.7875	0.2502	0.8179
		O	0.2003	0.5472	0.2927	0.6925
	G2	X	0.0493	0.6115	0.0578	0.6664
		O	0.2797	0.6181	0.2335	1.1690
Platelet	G1	X	0.2981	0.7925	0.3428	0.7943
		O	0.0279	0.5758	0.0245	0.5627
	G2	X	0.0625	0.6241	0.0818	0.6781
		O	1	0.3078	1.0000	2.3898
Fresh Frozen Plasma	G1	X	0.3024	0.8168	0.3057	0.8375
		O	0.0003	0.3254	0.0020	3.1800
	G2	X	0.0654	0.6286	0.0286	0.6626
		O	-	0.1226	-	5.4528
Cryoprecipitate	G1	X	0.3073	0.7779	0.3104	0.8041
		O	-	-	-	-
	G2	X	0.0473	0.6542	0.0654	0.6412
		O	-	-	-	-
